# House officer procedure documentation using a personal digital assistant: a longitudinal study

**DOI:** 10.1186/1472-6947-6-5

**Published:** 2006-01-26

**Authors:** Steven B Bird, David R Lane

**Affiliations:** 1 Department of Emergency Medicine, University of Massachusetts Medical School, Worcester, Massachusetts, USA

## Abstract

**Background:**

Personal Digital Assistants (PDAs) have been integrated into daily practice for many emergency physicians and house officers. Few objective data exist that quantify the effect of PDAs on documentation. The objective of this study was to determine whether use of a PDA would improve emergency medicine house officer documentation of procedures and patient resuscitations.

**Methods:**

Twelve first-year Emergency Medicine (EM) residents were provided a Palm V (Palm, Inc., Santa Clara, California, USA) PDA. A customizable patient procedure and encounter program was constructed and loaded into each PDA. Residents were instructed to enter information on patients who had any of 20 procedures performed, were deemed clinically unstable, or on whom follow-up was obtained. These data were downloaded to the residency coordinator's desktop computer on a weekly basis for 36 months. The mean number of procedures and encounters performed per resident over a three year period were then compared with those of 12 historical controls from a previous residency class that had recorded the same information using a handwritten card system for 36 months. Means of both groups were compared a two-tailed Student's t test with a Bonferroni correction for multiple comparisons. One hundred randomly selected entries from both the PDA and handwritten groups were reviewed for completeness. Another group of 11 residents who had used both handwritten and PDA procedure logs for one year each were asked to complete a questionnaire regarding their satisfaction with the PDA system.

**Results:**

Mean documentation of three procedures significantly increased in the PDA vs handwritten groups: conscious sedation 24.0 vs 0.03 (p = 0.001); thoracentesis 3.0 vs 0.0 (p = 0.001); and ED ultrasound 24.5 vs. 0.0 (p = 0.001). In the handwritten cohort, only the number of cardioversions/defibrillations (26.5 vs 11.5) was statistically increased (p = 0.001). Of the PDA entries, 100% were entered completely, compared to only 91% of the handwritten group, including 4% that were illegible. 10 of 11 questioned residents preferred the PDA procedure log to a handwritten log (mean ± SD Likert-scale score of 1.6 ± 0.9).

**Conclusion:**

Overall use of a PDA did not significantly change EM resident procedure or patient resuscitation documentation when used over a three-year period. Statistically significant differences between the handwritten and PDA groups likely represent alterations in the standard of ED care over time. Residents overwhelmingly preferred the PDA procedure log to a handwritten log and more entries are complete using the PDA. These favorable comparisons and the numerous other uses of PDAs may make them an attractive alternative for resident documentation.

## Background

An U.S. Institute of Medicine report estimates 44,000 to 98,000 hospitalized patient deaths are due to medical errors [1]. This has provided an impetus to standardize measures of performance. In fact, a recent U.S. National Academy of Sciences report suggests that government programs should reward high-quality health care by paying higher fees or bonuses to the best doctors, hospitals, and nursing homes [2]. In an environment demanding comparative measures of quality of care and physician and hospital performance, objective evidence of physician competence could become increasingly important in licensing, hiring, privileging, and promotion processes. Additionally, the impact of such performance-based credentialing on academic medicine and physicians-in-training has yet to be determined.

Documentation of physician competence begins during post-graduate medical training. The U.S. Residency Review Committee (RRC) for Emergency Medicine (EM) requires that residency programs document house officers' experience with invasive procedures and major resuscitations [3]. Historically, documentation of procedures and major resuscitations was achieved with handwritten logbooks or index card systems. In the last several years, several residency programs have instituted web-based documentation programs, and more recently, programs have introduced personal digital assistants (PDA) to store procedural and other data, replacing traditional handwritten index cards and logbooks [4-6]. PDAs are small hand-held computers whose portability and memory capacity have made them valuable in many aspects of health care, including research, education, documentation, drug prescriptions, patient tracking, online medical literature access, and daily reference [4,5,7-16].

We have previously reported first-year resident experience using a PDA for documentation [4]. This study expands that work by comparing the cumulative three-year experience of residents using a PDA for all procedural documentation with the three-year experience of residents using a traditional handwritten index card system. We also sought to obtain a qualitative evaluation of the PDA system from a group of residents who had used both paper-based and PDA-based procedure logs for one year.

## Methods

### Study design

The study design, setting, and protocol were previously reported [4]. This was a prospective study involving a retrospective cohort. The study analyzes EM resident documentation of procedures, patient resuscitations and patient follow-ups throughout their three-year residency. To determine the completeness of log entries by both documentation methods, a randomly selected group of 100 procedure log entries from each cohort were analyzed by two reviewers. Entries were classified as either "complete", "complete but illegible", or "incomplete." If the two reviewers could not agree on the completeness or legibility, a third reviewer adjudicated.

To determine resident physician satisfaction with the PDA procedure log, a group of residents that had used a handwritten procedure log for one year and the PDA procedure log for one year were surveyed using a 5-point Likert scale (1 = strongly agree, 5 = strongly disagree).

These studies were considered exempt from review by the Institutional Review Board of the University of Massachusetts Medical School.

### Study setting and population

Prospective data were collected from the emergency medicine residency at the University of Massachusetts Medical Center (UMMC) in Worcester, Massachusetts, USA, from July 1999 to June 2002. The EM residency is an RRC accredited three-year program with 12 residents per year. UMMC is a Level I trauma center with 365 in-patient beds and an annual emergency department (ED) census of 75,000 visits. The secondary training site has an ED volume of 47,000 emergency visits per year and 299 in-patient beds.

### Study protocol

Upon beginning residency training, each resident was provided a PalmV PDA (Palm, Inc., Santa Clara, California, USA). A customizable data collection application was constructed using Pendragon Forms 3.0 (Pendragon Software Corporation, Libertyville, Illinois, USA. ), and installed into each PDA. The Pendragon Forms input key was designed to fit on a single PDA screen. The PDA version closely resembled the handwritten version and both included a free-text or "write-in" area to be used at the residents' discretion. The prospective cohort consisted of 12 residents undergoing training. Using the Palm V's handwriting recognition capability and customized pull down menus, residents were required to enter the specific rotation, date, patient's initials, six-digit medical record number, age, and diagnosis. Then, from pull-down menus, the following data were entered: procedures performed (up to four per patient); follow-up call ('yes' or 'no'); and patient status ('stable, 'unstable adult medical', 'unstable adult surgical', unstable pediatric medical', or 'unstable pediatric surgical'). Residents were instructed to enter pertinent information as soon as possible following the procedure or resuscitation. The PDA form mandated that for each procedure log entry the patient's medical record number, initials, age, patient status, and diagnosis be entered. If the resident did not input any of these, the program would not save the entry. The residents were instructed to download (or "hot-sync") the data from their PalmV to the residency coordinator's desktop computer on a weekly basis. The synchronization process takes approximately 30 seconds per PDA. Documentation from 36 monthly rotations per resident was collected.

The retrospective cohort consisted of 12 residents undergoing training during the previous 3 years. This group was required to track 20 procedures, four types of resuscitations, and patient follow-ups using a traditional handwritten index card. These handwritten data were then transcribed into a computer database by the residency coordinator.

Using a 5-point Likert scale, another group of residents who had used both the handwritten procedure log and the PDA-based instrument for one year were asked if they preferred the PDA procedure log, if they preferred the paper-based log, and if they would recommend going back to the handwritten system. Additionally, they were asked to estimate how much time it takes to make a single entry in both the handwritten and PDA versions. Lastly, the residents were asked to estimate what percentage of procedures they entered using both procedure log types.

### Data analysis

We analyzed differences in procedure documentation between the PDA form and handwritten index cards based on the 20 procedures that were present on both platforms. These data are presented in Table 1. For the prospective study group, nasotracheal and endotracheal intubations were combined, and their sum is listed as endotracheal intubations. Data from the PDA and retrospective cohort groups were entered into GB STAT (Dynamic Microsystems, Inc., Silver Spring, Maryland, USA). Data from the retrospective cohort were combined with means and standard errors calculated for all measurements. Means are representative of the number of procedures, unstable patients, and follow-ups per resident for the entire three-year residency. The cohort and study group means were compared using a two-tailed Student's t test with a Bonferroni correction for multiple comparisons. Maintaining an alpha error of 0.05, the upper limit of statistical significance is therefore p = 0.002, rather than 0.05.

**Table 1:** Mean number of procedures and specific patient encounters during three-year residency as documented by handwritten versus PDA method.

**Procedure**	**Handwritten Cohort**	**PDA cohort**	**P value**
Arthrocentesis	2.5	2.0	0.48
Cardioversion/Defibrillation	26.5	11.5	0.001
Central line	72.0	76.0	0.76
Chest tube	17.5	15.5	0.18
Conscious sedation	0.03	24.0	0.001
Cricothyrotomy	2.0	0.5	0.16
Dislocation	16.0	13.0	0.20
DPL	2.0	2.5	0.63
Endotracheal intubation	115.5	102.0	0.18
Fracture reduction/ Splint	64.5	56.5	0.38
Internal pacing	5.5	4.0	0.34
Laceration repair	180.5	149.5	0.06
Lumbar puncture	39.5	33.0	0.20
Nasal packing	7.5	4.5	0.67
Open chest cardiac massage	1.0	0.5	0.51
Pericardiocentesis	2.5	2.0	0.63
Thoracentesis	0.0	3.0	0.001
Ultrasound	0.0	24.5	0.001
Vaginal delivery	26	31	0.10
Unstable adult medical	509.5	418.5	0.13
Unstable adult surgical	230.5	87.5	0.14
Unstable pediatric medical	117.0	35.5	0.01
Unstable pediatric surgical	34.0	20.0	0.18
Patient Follow-up	29.5	57.0	0.59

The means and standard errors for the residents' responses to the questionnaire were calculated using GB STAT.

## Results

Mean documentation of all procedures, patient encounters, and patient follow-up are provided in Table 1. Three procedures significantly increased in the PDA vs handwritten groups: conscious sedation 24.0 vs. 0.03 (p = 0.001); thoracentesis 3.0 vs 0.0 (p = 0.001); and ED ultrasound 24.5 vs 0.0 (p = 0.001). Only the number of cardioversions/defibrillations documented was increased significantly in the handwritten group: 26.5 vs 11.5 (p = 0.001). Complete data are shown in Table 1.

All eleven residents returned the anonymous surveys. Residents who had used both the handwritten and PDA-based procedure log overwhelmingly preferred the PDA-version (mean Likert scale score of 1.6 ± 0.9). When asked if they would prefer to return to a paper-based system, the residents overwhelmingly replied in the negative (mean Likert scale score of 4.5 ± 0.8). The estimated mean time to record a single procedure entry was 30 ± 8 seconds with the paper-based log and 34 ± 8 seconds with the PDA. Of the 100 PDA entries examined, 100% were entered completely. In the handwritten group only 91% were complete, including 4% that were illegible.

## Discussion

This study is a summary of the full three-year residency experience of EM residents using PDAs for procedure tracking. A gold standard for procedural documentation should combine efficiency, accuracy, accessibility of results, and portability with low cost. Traditional logbook and index card documentation methods are eclipsed in a number of these categories by newer PDA documentation methods, although the PDAs are not without limitations.

Elimination of secretarial or resident transcription of procedures from individual paper forms to a computerized database represents a significant improvement in PDA documentation. Removing this step improves accuracy of the data by eliminating transcription errors, and improves efficiency, leading to timelier access to results. Perhaps the most significant advantage of PDAs is the additional software that can be provided to resident physicians. Invaluable reference tools such as pharmaceutical programs, clinical reference materials, and medical calculators, give residents a wealth of information within easy reach. Use of a PDA may also have tangible advantages to patient care. Grasso and Genest found that medication error rates might be reduced by physician use of PDA pharmaceutical resources [12]. As medication errors are known to be a significant cause of morbidity and increased hospitalization costs [1], a modest reduction in medication ordering errors with PDA use should provide hospitals and residency programs with sufficient incentive to invest in the technology. The combination of these benefits would appear to make PDAs more attractive to program directors and residents.

The cost of implementing a PDA documentation system is determined by the purchase of the PDAs as well as the proprietary software with which to enter and track the data. Typical cost for a PDA is roughly $200–$400 USD. License fees for proprietary software are more variable, but can be expected to cost $25–$100 USD per resident per year. The cost per resident in this study was approximately $50 USD. Cost savings on transcription and secretarial time using a PDA system may offset the initial expenses of the PDAs and software. A previous study has shown that the costs of PDA and handwritten systems are nearly comparable [9]. As more computer-savvy physicians gain experience in PDA software design, use of institution-specific, non-proprietary software that does not require license fees may be an option.

Continuing advances in PDA hardware and software offer further potential improvements in handheld applications for physicians and other healthcare providers. Larger screens with better definition may allow patients to complete surveys and other data-entry forms free from traditional paper-based systems. Additionally, improved battery life and PDA durability has allowed use of these devices in the developing world, including at least clinical trials using bedside randomization in Sri Lanka [17]. Improved built-in wireless network capabilities will enable clinicians to more rapidly access data on the Internet and institutional networks, allowing true real-time and bedside literature searches and acces to patient laboratory and radiographic data. It would appear, therefore, that we are still in the nascent stages of clinical PDA use.

Our results suggest that use of a PDA did not significantly change resident procedure or patient resuscitation documentation over a three-year period. Statistically significant differences between the prospective and retrospective cohorts likely represent alterations in the standard of care over time. That is, the increase in ultrasound use reflects the routine, daily usage of emergency department-based ultrasound imaging. While the handwritten cohort intermittently did have access to an ultrasound machine, it was frequently broken and no formal instruction in its use was available to the residents or attending physicians. The increase in thoracentesis is also likely a reflection of changes in ED care. As patients wait longer for an available bed on the hospital wards, procedures formerly reserved until admission are more commonly performed in the ED. In addition, with the availability of ED ultrasound, thoracentesis can more easily be performed in the ED as opposed to in the radiology department or on the medical ward with ultrasound assistance. There is no readily evident reason why statistically more conscious sedations were documented in the PDA cohort. Regardless, the favorable documentation comparison and the numerous other benefits of PDAs may make them an attractive alternative for resident documentation.

Comparing a prospective cohort to a retrospective cohort creates potential for error in several ways. For example, residents from the retrospective cohort may have documented at a lower or higher rate than the more recent class. There is no systematic bias to this study and no way to reasonably account for this type of error. The number of procedures available to the residents may also have changed over time. From the beginning of the handwritten cohort to the end of the PDA cohort the University of Massachusetts Medical Center Emergency Department annual census increased by approximately 30%. This may not represent a true 30% increase in procedure availability, however, as changes in the number of residents staffing the ED each day during the time of the two cohorts increased by roughly 20%. Thus, if ED volume increased by 30% and resident staffing by 20%, there could be a slight bias towards more patient encounters in the PDA cohort. However, we have previously determined the number of patient encounters per resident per day over time, and found that there was no significant change over several years (data not shown).

Assessing the accuracy of resident documentation is a difficult task [18,19]. Accuracy of procedural documentation is largely dependent on resident compliance, which may equally affect paper or PDA documentation methods [4]. This study was not intended to address resident documentation compliance. In the future, hospital-wide wireless computer networks may allow residency directors to identify missed documentation or procedures, and thus easily determine resident compliance.

Perhaps the most important question in resident education is what constitutes competence? Some authors have previously sought to quantify competence [20,21], but no consensus exists on how to best determine a resident's clinical or procedural competence. Development of more sophisticated PDA software may allow confidential attending physician comments regarding the competence of a particular encounter or skill. While such a PDA program may be useful, the quantification of physician competence is difficult and beyond the scope of this study.

## Conclusion

Use of a personal digital assistant (PDA) during a three-year residency did not significantly change resident procedural documentation compared to a traditional handwritten system. The numerous additional features and conveniences of PDAs may make them an attractive documentation alternative for residency programs.

## Competing interests

The author(s) declare that they have no competing interests.

## Authors' contributions

SB conceived of the study, assisted in the design of the PDA software, assisted in data analysis, and edited the manuscript. DL assisted in data analysis and drafted the manuscript. All authors read and approved the final manuscript.

**Figure 1:**
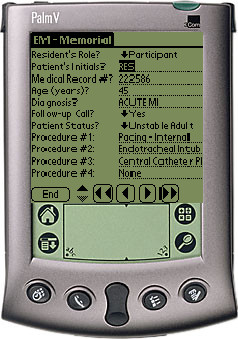
Screenshot of the PDA procedure documentation data entry screen.

## Pre-publication history

The pre-publication history for this paper can be accessed here:


